# Exogenous Supplementation with DAO Enzyme in Women with Fibromyalgia: A Double-Blind Placebo-Controlled Clinical Trial

**DOI:** 10.3390/jcm12206449

**Published:** 2023-10-10

**Authors:** Gülşah Okutan, Guerthy Melissa Sánchez Niño, Ana Terrén Lora, Sara López Oliva, Ismael San Mauro Martín

**Affiliations:** Research Centers in Nutrition and Health (CINUSA Group), Paseo de la Habana 43, 28036 Madrid, Spain; investigacion@grupocinusa.es (G.O.); melissa@grupocinusa.es (G.M.S.N.); ana.terren@grupocinusa.es (A.T.L.); sara@grupocinusa.es (S.L.O.)

**Keywords:** fibromyalgia, diamine oxidase, DAO supplementation, histamine intolerance

## Abstract

Fibromyalgia (FM) is characterized by chronic musculoskeletal pain, muscle tension, joint mobility loss, and several psychological symptoms severely affecting patient well-being. Histamine is naturally degraded in the small intestine by diamine oxidase (DAO). Hereditary or acquired DAO deficiency causes extracellular histamine accumulation, leading to symptoms similar to those of individuals diagnosed with FM. Thus, this study aimed to assess the efficacy of adding DAO supplementation for 8 weeks to their standard therapy. We randomly assigned 100 women with FM (age: 33–61 years) to the supplementation and control groups. The Fibromyalgia Impact Questionnaire (FIQ), the Pain Catastrophizing Scale (PCS), and intensity scales were applied for a series of clinical symptoms together with the Bristol scale to assess the added value of DAO supplementation. Patients in both groups were receiving complete pharmacological support but some differences in the number of subjects receiving analgesics, antidepressants, and anxiolytics was noted. Patients in both study groups experienced favorable changes during the evaluation period as indicated by their final FIQ and PCS scores, particularly in the DAO group in the latter questionnaire. Qualitatively, the patients assigned to the DAO treatment group had lower scores for fatigue, anxiety, depression, burning and for rumination, magnification, and helplessness.

## 1. Introduction

Fibromyalgia (FM) is a syndrome characterized by the presence of chronic pain in the musculoskeletal system, as well as muscle tension, joint mobility loss, sleep problems, fatigue, mood disorders, cognitive dysfunction, anxiety, depression, and extreme sensitivity, which result in an inability to carry out daily life activities [1,2]. FM has a prevalence of approximately 5% worldwide, most frequently affecting women, who start presenting symptoms between 30 and 35 years of age [3]. However, despite its high prevalence, it remains a poorly understood condition and a challenge to diagnose accurately. A causal study has suggested that the mast cells presenting in the thalamus can participate in the development of inflammation and pain by secreting various neurosensitizing molecules, including histamine, IL-1β, IL-6, and TNF [4]. Approximately 90% of individuals suffering from FM resort to complementary treatments to alleviate their symptoms [5]. Moreover, non-pharmacological treatments have a higher acceptance compared to pharmacological treatments; however, their effectiveness is rated in a similar manner [6].

Currently, there is an increasing interest in the investigation of the possible relationship between FM and food-related issues, including food intolerances. In fact, several relationships have been observed between eating habits and the improvement of several FM symptoms [7]. Histamine is one of the molecules investigated for its relationship with FM symptoms, and it is naturally degraded mostly in the small intestine by diamine oxidase (DAO). Hereditary or acquired DAO deficiency causes an accumulation of extracellular histamine [8], which can lead to typical symptoms of histamine intolerance (HIT), including swelling, headache, hives, eczema, and gastrointestinal (GI) disorders [9]. These symptoms are also associated with other conditions and diseases and are generally caused by the ingestion of histamine-rich foods, alcohol, and drugs that release histamine or block DAO. The prevalence of HIT has been underestimated for decades, and its symptoms have been poorly understood [10,11,12]. The main cause of HIT is an alteration in the enzymatic degradation of histamine resulting from a genetic or acquired alteration of the enzymatic function of DAO, since GI diseases affecting enterocytes can also cause a decrease in DAO production [13].

According to the International Society of DAO Deficiency, the adverse effects of DAO deficiency include nasal congestion, asthma, hypotension, hypertension, arrhythmias, migraine, headaches, irritable bowel syndrome, constipation, satiety, stomach pain, vomiting, and muscle pain [14]. Several of these symptoms are also present in individuals with FM. 

Histamine intolerance due to a DAO deficiency may have a genetic, pathological, or pharmacological origin. Regarding genetic origin, some SNPs affecting DAO enzyme functionality (especially in Caucasian individuals) are rs10156191, rs1049742, rs2268999, and rs1049793. On the other hand, there are known pathologies that affect mucosal integrity result in altered DAO activity. Finally, substances such as biogenic amines, alcohol, and some drugs (chloroquine, clavulanic acid, cefuroxime, amitriptyline, etc.) could also cause DAO deficiency [15].

In this context, a recent study investigated the frequency of genetic DAO deficiency in Spanish women with FM based on four variations of the AOC1 gene (rs10156191, rs1049742, rs1049793, and rs2052129) and found that approximately 75% of the patients had at least one risk allele associated with DAO deficiency [16]. Another study revealed how oral DAO supplementation before meals could help improve HIT-related symptoms, including bloating, postprandial fullness, and abdominal pain, and their intensity [17].

Since no approved and effective drugs for FM are available in the market and individuals with HIT and FM share several symptoms, the objective of this study was to assess the efficacy of adding DAO supplementation to the standard treatment in women with FM for 8 weeks.

## 2. Materials and Methods

### 2.1. Design

**Figure 1:**
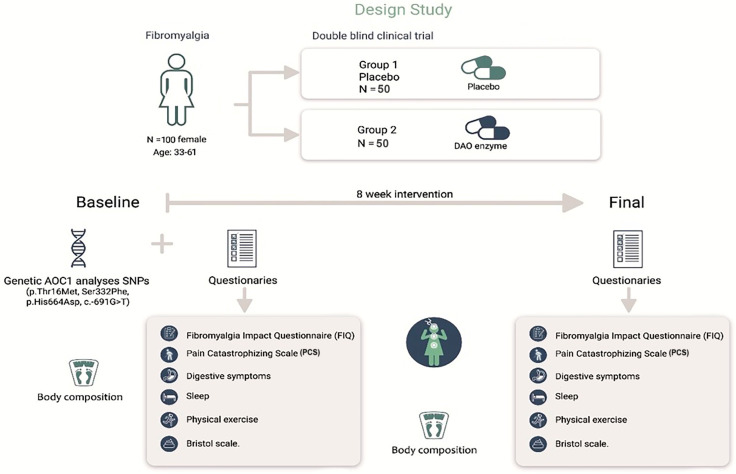
Trial design.

A double-blind, randomized, placebo-controlled clinical trial was conducted. (Figure 1).

### 2.2. Sample

A sample of 100 women with FM, aged 33–61 years (48.48 ± 7.35 years), were randomly assigned to treatment and control groups (Table 1). 

Age group between 30–61 years and medical diagnosis of FM were considered inclusion criteria. Exclusion criteria were multiple chemical sensitivities, following a low-histamine diet, treatment with DAO supplements, and some chronic diseases (cancer, AIDS, and Alzheimer’s disease).

### 2.3. Intervention

Subject were randomly assigned to one of the two groups: DAO group: gastro-resistant cellulose microcrystalline and hydroxypropylcellulose tablets with 4.2 mg of pig kidney protein extract rich in DAO and excipients for tableting and coating for gastro-protection; Placebo group: gastro-resistant cellulose microcrystalline and hydroxypropylcellulose tablets with the same form, aspect, and weight than the DAO tablets but without active dietary ingredient (only excipients). During intervention, participants took three tablets of the product per day with water, 20 min before main meals.

Due to intervention in some previous studies [18,19] having been performed for one month, with positive trends, longer treatment period was decided on in order to obtain a significant improvement.

### 2.4. Fibromyalgia Impact Questionnaire (FIQ)

The FIQ is a 10-item self-administered questionnaire, and it has been adapted into Spanish [20]. The first item (physical function) consists of 11 sub-items with four levels on a Likert scale. In item 2 (feeling good), patients indicate the number of days they have felt good during the previous week. Items 3 (absence from work) and 4 (work) refer to the number of missed days of work in the previous week and work difficulty, respectively. The remaining six items are scored using visual analogue scales (VAS), as in item 4, and their content assesses pain, fatigue, stiffness, anxiety, and depression.

The scores obtained in those items were adapted, recorded, and standardized to 10. The total numerical score is the sum of the scores obtained in each item, and the highest score represents a negative health status.

### 2.5. Pain Catastrophizing Scale (PCS)

The PCS is a 13-item self-administered scale that has been validated in Spanish in patients with fibromyalgia [21]. Each item consists of a statement about the feelings and thoughts related to pain, scored from 0 (never) to 4 (always) according to the degree of occurrence of the statement. It consists of three subscale scores assessing rumination, magnification, and hopelessness.

### 2.6. Clinical Symptoms

A series of clinical symptoms related to sleep quality, atopic dermatitis (dry skin, hives, and eczema), migraine, and, most importantly, GI disorders (bloating, abdominal pain, burning, nausea, vomiting, and flatulence) were assessed together with the Bristol scale (designed to assess the stool consistency). The questionnaires were self-administered to indicate intensity (0–10), where 0 indicated absence of symptoms and 10 indicated maximum intensity.

### 2.7. Statistical Analysis

Linear mixed models were used with patients as random effects, treatment and time as fixed effects, and the covariates, namely age, BMI, and baseline scores, centered on the analyzed response variable. For parameter estimation, we selected the restricted maximum likelihood approach, which has the advantage of including patients who attended the second visit, since this approach uses information from other similar cases that have complete data. Additionally, compound symmetry was selected for the covariance structure. The assumptions of the statistical models were relaxed given the large sample size used in the study.

On the other hand, the Mann–Whitney (M-W) test was used to compare the number of risk alleles in responding and non-responding patients, and the unpaired *t*-test was used to compare concomitant medication according to the study group or M-W if the distribution normality was seriously affected.

All contrasts were two-sided with a 95% confidence level using IBM SPSS Statistics 25.0 (IBM Corporation, Armonk, NY, USA).

## 3. Results 

### 3.1. Impact of Fibromyalgia and Pain Catastrophizing

First, we assessed the changes in the estimated means of the items of the FIQ and PCS questionnaires, as well as the total score in each case (Table 2), using linear mixed models with time, study group, and interaction as fixed effects (Table S1).

**Figure 2:**
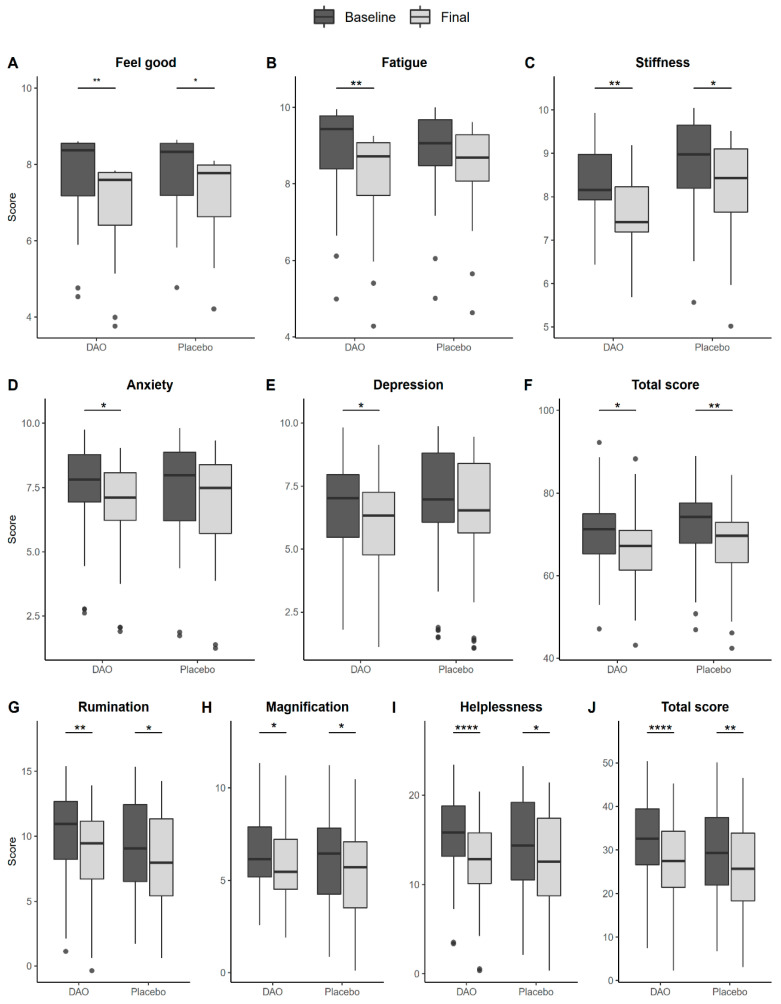
Predicted FIQ and PCS scores by study group according to time. (**A**): Changes in Feel good between subjects treated with DAO vs placebo pre- and post-intervention; (**B**): A: Changes in Fatigue between subjects treated with DAO vs placebo pre- and post-intervention; (**C**): Changes in Stiffness between subjects treated with DAO vs placebo pre- and post-intervention; (**D**): Changes in Anxiety between subjects treated with DAO vs placebo pre- and post-intervention; (**E**): Changes in Depression between subjects treated with DAO vs placebo pre- and post-intervention; (**F**): Changes in Total score between subjects treated with DAO vs placebo pre- and post-intervention; (**G**): Changes in Rumination between subjects treated with DAO vs placebo pre- and post-intervention; (**H**): Changes in Magnification between subjects treated with DAO vs placebo pre- and post-intervention; (**I**): Changes in Helplessness between subjects treated with DAO vs placebo pre- and post-intervention; (**J**): Changes in Total Score between subjects treated with DAO vs placebo pre- and post-intervention. The significance is presented after applying linear mixed models incorporating age, BMI, and centered scores for each response variable as covariates. * *p* < 0.05; ** *p* < 0.01; and **** *p* < 0.0001.

The results of the FIQ suggest that patients in the DAO group felt better (Figure 2A), and experienced less fatigue (Figure 2B), stiffness (Figure 2C), anxiety (Figure 2D), and depression (Figure 2E) after eight weeks of treatment. These changes were associated with a significant decrease in the total score (Figure 2F). However, the scores did not decrease sufficiently to show significant differences with respect to the scores of participants in the placebo group on these items or on the total score. Furthermore, the placebo group also reported feeling better (Figure 2A) and less stiff (Figure 2C), with a significant final total score (Figure 2F), largely due to the effect of other items that reduced the final scores, although without reaching significance.

The dimensions of the PCS and the resulting total score were also analyzed. In this case, the patients assigned to the DAO treatment group had lower scores for rumination (Figure 2G), magnification (Figure 2H), and, especially, helplessness (Figure 2I). Consequently, the final score for the PCS was better in this group than in the placebo group (Figure 2J). However, no significant differences were found between the two groups in any dimension.

### 3.2. Clinical Symptoms

A series of clinical symptoms related to sleep quality, atopic dermatitis, migraine, and GI disorders were assessed to determine whether they would cease to occur in participants after treatment (Table 3).

**Figure 3:**
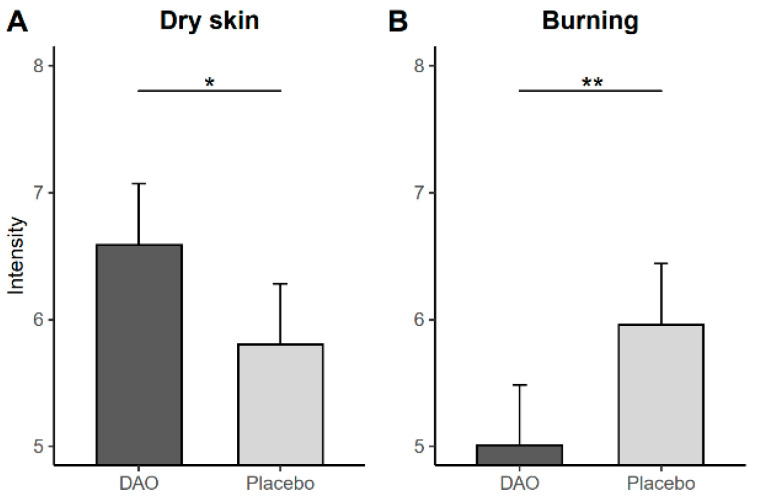
Intensity of clinical symptoms after treatment according to the study group. (**A**): Changes in Dry skin intensity between subjects treated with DAO vs placebo; (**B**): Changes in Burning intensity between subjects treated with DAO vs placebo. The estimated means and 95% confidence intervals are presented after applying a linear mixed model incorporating age, BMI, and centered scores of each response variable as covariates. * *p* < 0.05; ** *p* < 0.01.

Regarding the symptoms related to atopic dermatitis, hives were observed to be less severe after the intervention in the placebo group. Furthermore, dry skin was also less severe, and the mean was lower than that in the DAO group (Figure 3A).

Regarding GI disorders, bloating and flatulence decreased in the placebo group; however, the mean did not differ between the study groups. In contrast, burning improved only in patients who took DAO (Figure 3B), whereas all other symptoms were largely unchanged during the last visit after treatment.

### 3.3. Responders

The results obtained using questionnaires and clinical symptom scales did not show a large improvement in patients assigned to the DAO group (Table S2). Thus, patients who exhibited improvements in the typical FM symptoms after treatment with DAO, as determined by the FIQ scores, were classified as *responders* in order to identify other symptoms that also improved. 

**Figure 4:**
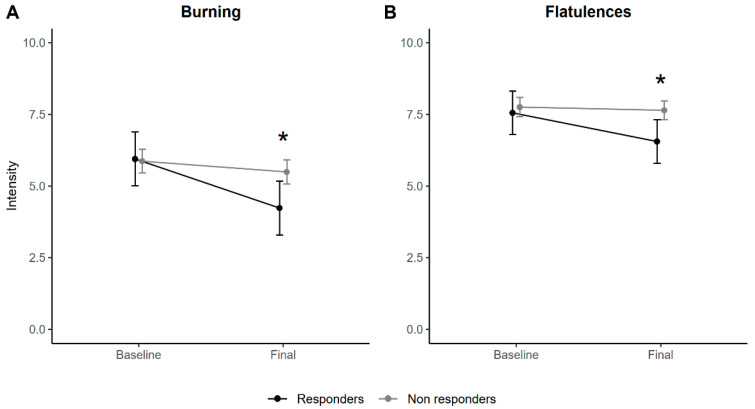
Estimated means for responders with a 20% improvement as assessed by the FIQ after eight weeks of treatment and non-responders according to the time. (**A**): Changes in Burning intensity between subjects treated with DAO vs placebo pre- and post-intervention; (**B**): Changes in Flatulences intensity between subjects treated with DAO vs placebo pre- and post-intervention. The estimated means and 95% confidence intervals are presented after applying linear mixed models incorporating age, BMI, and centered scores of each response variable as covariates. * *p* < 0.05.

Given the sample sizes, a 20% improvement in the FIQ scores after treatment was proposed, with 7 *responders* (16.28%) and 36 *non-responders* (83.72%) among those patients who attended the second visit. Thus, performing the same tests on patients assigned to the DAO group showed that both burning (Figure 4A) and flatulence (Figure 4B) improved in *responders* compared to those in *non-responders*. No significant changes in the rest of the symptoms were observed (Table S3).

Lastly, no more risk alleles associated with DAO deficiency were observed in any of the groups (2 ± 2.24 vs. 1.89 ± 1.60; M-W: *p* > 0.500). This fact highlights that the variants do not seem to be behind the effect that it had among the responders.

### 3.4. Concomitant Medication

Subjects in both groups continued receiving the regular medication including anxiolytics, anti-depressants, analgesics, etc. Patients in the group receiving DAO were already under treatment with an average of 7.8 ± 4.3 drugs and 1.85 ± 2.46 dietary supplements, while patients under placebo were regularly receiving an average of 6.26 ± 3.67 and 1.53 ± 2.3, respectively.

Even with a such therapeutic scheme, patients in the DAO group had PCS scores above 30 in the baseline, which are known to be clinically relevant. Such scores went down below the 30 thresholds during the supplementation period. In the placebo group, scores were already below 30 at baseline.

If we look at the drug groups, antidepressants and sedatives stood out for their higher consumption in the DAO group (1.10 ± 0.93 vs. 0.74 ± 0.86; unpaired *t*-test: *p* < 0.100), especially sedatives (1.23 ± 1.29 vs. 0.63 ± 0.91; unpaired *t*-test: *p* < 0.05), whose difference was significant. In addition, it was found that concomitant consumption of antidepressants, anxiolytics, and/or sedatives was significantly higher in the DAO group (DAO: 1.80 ± 1.49; Placebo: 1.11 ± 1.23; unpaired *t*-test: *p* < 0.05). Regarding the intake of antidepressants, it is relevant to highlight that, despite the differences between the groups, in the DAO group, the improvement of this specific item showed a significant difference (*p* = 0.030) when compared with baseline.

As for the significant changes observed in the placebo group after the intervention (Table 3), specifically bloating and flatulence could be due to the effect of the medication, since the placebo group was taking more drugs to treat swelling (0.82 ± 0.73 vs. 0.63 ± 0.67; M-W: *p* < 0.300) and the daily dose was significantly higher with respect to the DAO group (281.09 ± 382.46 vs. 136.38 ± 420.36; M-W: *p* < 0.05). The same observation was made for the number of drugs against flatulence (0.18 ± 0.46 vs. 0.05 ± 0.22; M-W: *p* < 150), although it is true that very few patients were treated against this ailment and their daily dose was highly variable in the placebo group (219.09 ± 905.05 vs. 3 ± 14; M-W: *p* = 0.200).

### 3.5. Stepwise Therapeutic Regimen

Another aspect to highlight and observed in the histories is that despite significant medication and supplementation, these patients have very high symptom scores. In itself, the recommendation of specific drugs could be interacting with each other and perhaps not having attended to an order of introduction, evaluation, and withdrawal or modification by therapeutic steps.

## 4. Discussion 

The objective of this study was to assess the efficacy of DAO supplementation for eight weeks in women with FM who were already under meaningful standard pharmacological treatment. Although patients who took DAO felt better, with less fatigue, stiffness, anxiety, and depression after the intervention, the effect was not sufficient to result in significant mean differences between the treatment and placebo groups. Moreover, the scores on some other items were slightly lower in the placebo group, although not significantly, and contributed to a decrease in the total score in this group.

Regarding the PCS, although the total score decreased in both groups, the effect was greater in those who took DAO. Several elements influence the magnitude of pain experienced in FM, including the number of affected body areas, psychological aspects, and the reaction to applied peripheral stimuli [22]. Importantly, performance-based and self-reported physical functions are not correlated in individuals with chronic musculoskeletal pain [23]. A study conducted with patients diagnosed with FM applying FIQ and PCS showed that they demonstrated a worse subjective performance in terms of their physical function, in contrast to their objective performance; additionally, subjective assessments were poor indicators of the physical functional status when patients with FM experienced high levels of catastrophizing [24]. This result is considered to be of clinical relevance, since subjective self-evaluation of health status has a great impact in the quality of life of these types of patients.

Considering the results of the placebo group for the questionnaires and according to the literature, the effect of pharmacological treatments over placebo in reducing the symptoms in patients with FM is small and is accompanied by relevant dropout rates due to adverse events. A review assessing 18 studies with 3546 placebo patients showed that the combined estimate of a 50% reduction in pain with placebo was 18.6% and the combined estimate of dropouts due to adverse events in the placebo groups was 10.9% [25]. This confirms that the important placebo effect observed in our study is common to other studies with this type of population.

DAO supplementation did not reduce the assessed clinical symptoms of sleep quality, migraine, and skin conditions. An experimental study conducted in patients with episodic migraine and DAO deficiency reported that the duration and number of migraines reduced after treatment with DAO [18]. However, the placebo group also experienced fewer attacks, and no differences were found between groups in any of the cases [18]. In contrast, DAO may be involved in the pathogenic cascade leading to skin rashes, and DAO supplementation may be effective in relieving symptoms [19]. Furthermore, several studies showed that reduced DAO levels were associated with symptoms typical of atopic dermatitis [26,27,28,29].

In the case of GI disorders, previous studies observed that DAO supplementation could improve bloating, diarrhea, abdominal pain, belching, and fullness [17]. Our study was able to confirm a decrease in burning in the DAO group. This relationship may be related to the significant inhibition of basal nocturnal gastric secretion, as well as acid secretion, which are stimulated by several agents, including histamine, pentagastrin, caffeine, insulin, sham feeding, and food, by binding to the H2 receptor and activating adenylate cyclase with cAMP generation, suggesting that histamine plays a physiological and molecular role in gastric acid secretion [30,31]. DAO can metabolize part of this histamine, blocking the production of the proton pump and preventing the pH from decreasing significantly, thereby reducing acidity or burning. The typical symptoms of patients with irritable bowel syndrome and FM have been extensively studied [32]; however, the effects of DAO supplementation on GI symptoms associated with FM had not been investigated.

Subjects supplemented with DAO enzyme obtained better results in anxiety and depression than in GI symptoms. This effect could be explained because histamine can act as an endogenous neurotransmitter that could act directly on neurons and neuronal structures [33] and therefore could be involucrate in anxiety [34] and depression [35], among others. On the other hand, other papers show a relationship between histamine and some gastrointestinal disorders, although we have not found great changes in this issue. The reasons for these differences could be that patients were treated with several drugs that could affect negatively to GI symptoms. In addition, the GI symptoms of FM may have a multicausal origin. Further, the placebo effect difficulted to find differences between groups, whose sample size was small. Several limitations could explain the limited statistical power observed in this study in favor of DAO supplementation in a population suffering from FM. Among the possible reasons, there were several limitations in the collection and control of extraneous variables are found. These include an excessive and varied regular consumption of drugs (such as anti-inflammatory, anxiolytics, antidepressants, and sedatives), which represent a real challenge in the data collection process. In addition, most of the consumed drugs could potentially interact with DAO functionality, having an effect on the supplementation effect. In fact, the similar structure of drugs cited above with histamine could explain their potential to bind to the active site of DAO and reduce its function [33]. Moreover, in our study, sedatives were overrepresented in the DAO group and these could attenuate the expected effect of the supplement, as well as antidepressants, that is, women with more depressive symptoms, which could explain a bias in the perception of the patients in the face of the subjective assessment of numerous symptoms. Nevertheless, a significant difference was observed in the depression item of the FIQ questionnaire (*p* < 0.030) only in the DAO group between the baseline and the final. Therefore, maybe a new approach to the stepwise therapeutic regime in FM patients should be considered in the future, adding DAO supplementation in the first stages benefiting from the safety profile of the natural enzyme and delaying the use of potent drugs with more potential side effects. Another important limitation is that FM is a complex disease, and its nature is not entirely known. The symptoms of FM, some of which are similar to those of patients with HIT, may have a different causal or even multicausal origin. Further, we observed a large placebo effect, which is easily manifested in this patient population, making it difficult to find differences between groups in the short term. Other limitations were the small sample size, or the absence of complementary analyzes such as the measurement of serum DAO or a low-histamine dietary intervention accompanying supplementation, which we hope will be the motivation for future research on this topic.

## 5. Conclusions

The effect of DAO enzyme supplementation in women with fibromyalgia seems to be notable on the outcome of the PCS questionnaire score, on fatigue, and on some digestive symptoms. We have described a potential treatment approach in women with fibromyalgia who respond positively to DAO deficiency supplementation. The effects obtained on top of a complete medication scheme are clinically relevant as they relate to consistent and clinically meaningful markers of disability in these patients. Based on the huge drug use in this disease, the quick changes in clinical symptoms observed in this clinical trial and the easy possibility of carrying out a screening and detection of HIT or DAO deficiency, the supplementation with exogenous DAO could be and potential therapeutic approach in fibromyalgia. Finally, conducting a double-blind, placebo-controlled trial with a crossover design, more sample and intervention time could be highly informative in the future.

## Figures and Tables

**Table 1:** Baseline characteristics.

	DAO (n = 50)	Placebo (n = 50)	Total
Age	48.48 ± 7.17	48.48 ± 7.60	48.48 ± 7.35
BMI (Kg/m^2^)	27.59 ± 5.08	27.65 ± 5.77	27.62 ± 5.41
Nationality, n (%)			
Spanish	45 (90)	48 (96)	93 (93)
Romanian	2 (4)	0 (0)	2 (2)
Ecuadorian	1 (2)	1 (2)	2 (2)
Peruvian	0 (0)	1 (2)	1 (1)
Bolivian	1 (2)	0 (0)	1 (1)
Japanese	1 (2)	0 (0)	1 (1)

**Table 2:** Mean change in questionnaire scores after treatment by study group.

		Final—Baseline DAO Group		Final—Baseline Placebo Group	
		Mean Change (95% CI)	*p*-Value	Mean Change (95% CI)	*p*-Value
FIQ					
	Physical function	−0.22 (−0.78, 0.34)	0.441	−0.25 (−0.82, 0.32)	0.390
	Feel good	−0.77 (−1.25, −0.30)	0.002	−0.56 (−1.03, −0.09)	0.021
	Work missed	1.13 (−0.23, 2.50)	0.100	0.70 (−0.62, 2.03)	0.288
	Job ability	−0.24 (−0.74, 0.27)	0.353	−0.37 (−0.87, 0.13)	0.146
	Pain	−0.22 (−0.67, 0.24)	0.348	−0.36 (−0.81, 0.09)	0.118
	Fatigue	−0.71 (−1.23, −0.19)	0.008	−0.40 (−0.91, 0.11)	0.125
	Morning tiredness	−0.43 (−0.95, 0.09)	0.107	−0.53 (−1.05, −0.01)	0.046
	Stiffness	−0.74 (−1.23, −0.26)	0.003	−0.55 (−1.03, −0.07)	0.026
	Anxiety	−0.71 (−1.27, −0.15)	0.014	−0.49 (−1.05, 0.74)	0.088
	Depression	−0.70 (−1.33, −0.07)	0.030	−0.42 (−1.05, 0.20)	0.182
	Total score	−3.97 (−7.07, −0.86)	0.013	−4.67 (−7.76, −1.59)	0.003
PCS					
	Rumination	−1.49 (−2.41, −0.57)	0.002	−1.10 (−2.01, −0.18)	0.020
	Magnification	−0.68 (−1.30, −0.05)	0.035	−0.75 (−1.37, −0.13)	0.019
	Helplessness	−3.01 (−4.26, −1.75)	<0.0001	−1.79 (−3.04, −0.55)	0.005
	Total score	−5.14 (−7.48, −2.79)	<0.0001	−3.63 (−5.96, −1.30)	0.003

Note: Changes in the estimated means are presented after incorporating age, BMI, and centered scores for each response variable as covariates. Changes with a negative sign denote a decrease after eight weeks of treatment.

**Table 3:** Mean change in the variables related to sleep, intensity of atopic dermatitis, migraine, and GI disorders by study group.

		Final—Baseline DAO Group		Final—Baseline Placebo Group	
		Mean Change (95% CI)	*p*-Value	Mean Change (95% CI)	*p*-Value
Sleep					
	Sleep quality	0.13 (−0.42, 0.68)	0.646	0.45 (−0.09, 1.00)	0.102
Atopic dermatitis					
	Dry skin	0.13 (−0.58, 0.85)	0.712	−0.62 (−1.33, −0.09)	0.086
	Hives	−0.34 (−1.15, 0.48)	0.419	−0.97 (−1.78, −0.16)	0.019
	Eczema	0.80 (−0.32, 1.92)	0.161	0.30 (−0.83, 1.43)	0.597
Migraine					
	Migraine	−0.24 (−1.04, 0.57)	0.562	−0.58 (−1.38, 0.22)	0.150
GI disorders					
	Bloating	−0.34 (−0.81, 0.12)	0.148	−0.66 (−1.13, −0.20)	0.005
	Abdominal pain	−0.22 (−0.80, 0.35)	0.443	−0.39 (−0.97, 0.19)	0.182
	Burning	−0.58 (−1.24, 0.08)	0.086	0.43 (−0.25, 1.10)	0.209
	Flatulence	−0.25 (−0.78, 0.29)	0.366	−0.60 (−1.14, 0.06)	0.030
	Bristol scale (1–7)	0.02 (−0.40, 0.40)	0.991	0.10 (−0.29, 0.50)	0.606

Note: Changes in the estimated means are presented after incorporating age, BMI, and centered scores for each response variable as covariates. Changes with a negative sign signify a decrease after eight weeks of treatment.

## Data Availability

The datasets generated during and/or analysed during the current study are available from the corresponding author on reasonable request.

## Supplementary Materials

The following supporting information can be downloaded at: https://www.mdpi.com/article/10.3390/jcm12206449/s1. Table S1: Fixed effects for linear mixed models examining changes in questionnaires; Table S2: Fixed effects for the mixed linear model examining the clinical symptoms; Table S3: Fixed effects for the mixed linear model examining the clinical symptoms according to responders.
