# Supplementary material for: Exogenous Supplementation with DAO Enzyme in Women with Fibromyalgia: A Double-Blind Placebo-Controlled Clinical Trial
Source: J Clin Med. 2023 Oct 10;12(20):6449. doi: 10.3390/jcm12206449 (PMC10607251; doi:10.3390/jcm12206449)
Supplement: Supplementary file 1 [file jcm-12-06449-s001.zip › jcm-2616282-supplementary.pdf]

*Article*

# Exogenous supplementation with DAO enzyme in women with fibromyalgia: a double-blind placebo-controlled clinical trial

## SUPPLEMENTARY INFORMATION

**Table S1.** Fixed effects for linear mixed models examining changes in questionnaires

| | | Estimate | SE | *t* | *P*-value | 95% CI |
|---|---|---|---|---|---|---|
| **FIQ** | | | | | | |
| | **Physical function** | | | | | |
| | Time | 0.22 | 0.28 | 0.77 | 0.441 | -0.34, 0.78 |
| | Group | 0.06 | 0.28 | 0.21 | 0.837 | -0.51, 0.62 |
| | Group x time | 0.03 | 0.40 | 0.07 | 0.942 | -0.77, 0.83 |
| | **Feel good** | | | | | |
| | Time | 0.77 | 0.24 | 3.22 | **0.002** | 0.30, 1.25 |
| | Group | 0.22 | 0.25 | 0.90 | 0.371 | -0.27, 0.71 |
| | Group x time | -0.21 | 0.34 | -0.63 | 0.533 | -0.88, 0.46 |
| | **Work missed** | | | | | |
| | Time | -1.13 | 0.67 | -1.70 | 0.100 | -2.50, 0.23 |
| | Group | -0.31 | 0.78 | -0.40 | 0.694 | -1.89, 1.27 |
| | Group x time | 0.43 | 0.94 | 0.46 | 0.650 | -1.48, 2.34 |
| | **Job ability** | | | | | |
| | Time | 0.24 | 0.25 | 0.93 | 0.353 | -0.27, 0.74 |
| | Group | -0.10 | 0.25 | -0.38 | 0.699 | -0.60, 0.40 |
| | Group x time | 0.13 | 0.36 | 0.37 | 0.711 | -0.57, 0.84 |
| | **Pain** | | | | | |
| | Time | 0.22 | 0.23 | 0.94 | 0.348 | -0.24, 0.67 |
| | Group | -0.13 | 0.23 | -0.57 | 0.571 | -0.59, 0.33 |
| | Group x time | 0.14 | 0.32 | 0.44 | 0.659 | -0.50, 0.79 |
| | **Fatigue** | | | | | |
| | Time | 0.71 | 0.26 | 2.72 | **0.008** | 0.19, 1.23 |
| | Group | 0.30 | 0.26 | 1.17 | 0.243 | -0.21, 0.82 |
| | Group x time | -0.31 | 0.37 | -0.84 | 0.403 | -1.04, 0.42 |
| | **Morning tiredness** | | | | | |
| | Time | 0.43 | 0.26 | 1.63 | 0.107 | -0.09, 0.95 |
| | Group | -0.07 | 0.26 | -0.27 | 0.784 | -0.59, 0.45 |

| | | | | | |
|---|---|---|---|---|---|
| Group x time | 0.10 | 0.37 | 0.27 | 0.789 | -0.64, 0.84 |
| **Stiffness** | | | | | |
| Time | 0.74 | 0.24 | 3.05 | **0.003** | 0.26, 1.23 |
| Group | 0.26 | 0.25 | 1.03 | 0.307 | -0.24, 0.75 |
| Group x time | -0.20 | 0.34 | -0.57 | 0.567 | -0.88, 0.48 |
| **Anxiety** | | | | | |
| Time | 0.71 | 0.28 | 2.50 | **0.014** | 0.14, 1.27 |
| Group | 0.23 | 0.29 | 0.81 | 0.418 | -0.33, 0.80 |
| Group x time | -0.22 | 0.40 | -0.56 | 0.579 | -1.02, 0.57 |
| **Depression** | | | | | |
| Time | 0.70 | 0.32 | 2.21 | **0.030** | 0.07, 1.33 |
| Group | 0.29 | 0.32 | 0.90 | 0.370 | -0.35, 0.92 |
| Group x time | -0.28 | 0.45 | -0.62 | 0.539 | -1.16, 0.61 |
| **Total Score** | | | | | |
| Time | 3.96 | 1.56 | 2.54 | **0.013** | 0.86, 7.07 |
| Group | -0.60 | 1.60 | -0.38 | 0.706 | -3.76, 2.55 |
| Group x time | 0.71 | 2.20 | 0.32 | 0.749 | -3.67, 5.08 |
| **PCS** | | | | | |
| **Rumination** | | | | | |
| Time | 1.49 | 0.47 | 3.20 | **0.002** | 0.57, 2.41 |
| Group | 0.28 | 0.47 | 0.59 | 0.553 | -0.65, 1.21 |
| Group x time | -0.40 | 0.66 | -0.60 | 0.549 | -1.70, 0.91 |
| **Magnification** | | | | | |
| Time | 0.68 | 0.32 | 2.14 | **0.035** | 0.05, 1.30 |
| Group | -0.14 | 0.32 | -0.46 | 0.649 | -0.77, 0.48 |
| Group x time | 0.07 | 0.45 | 0.17 | 0.870 | -0.81, 0.96 |
| **Helplessness** | | | | | |
| Time | 3.01 | 0.63 | 4.75 | **< 0.0001** | 1.75, 4.26 |
| Group | 1.11 | 0.64 | 1.74 | 0.084 | -0.15, 2.38 |
| Group x time | -1.21 | 0.89 | -1.36 | 0.177 | -2.98, 0.56 |
| **Total score** | | | | | |
| Time | 5.13 | 1.18 | 4.34 | **< 0.0001** | 2.79, 7.48 |
| Group | 1.29 | 1.21 | 1.07 | 0.285 | -1.08, 3.67 |
| Group x time | -1.51 | 1.67 | -0.91 | 0.367 | -4.81, 1.80 |

Note: Group coded as 0 = Placebo, 1 = DAO.

**Table S2.** Fixed effects for the mixed linear model examining the clinical symptoms

| | | Estimate | SE | *t* | *P*-value | 95% CI |
|---|---|---|---|---|---|---|
| Sleep | | | | | | |
| | Sleep quality | | | | | |
| | Time | -0.13 | 0.28 | -0.46 | 0.646 | -0.68, 0.42 |
| | Group | 0.24 | 0.26 | 0.90 | 0.368 | -0.28, 0.76 |
| | Group x time | -0.33 | 0.39 | -0.84 | 0.404 | -1.10, 0.45 |
| Atopic dermatitis | | | | | | |
| | Dry skin | | | | | |
| | Time | -0.13 | 0.37 | -0.37 | 0.712 | -0.84, 0.58 |
| | Group | -0.78 | 0.35 | -2.27 | 0.025 | -1.46, -0.10 |
| | Group x time | 0.75 | 0.51 | 1.49 | 0.140 | -0.25, 1.76 |
| | Hives | | | | | |
| | Time | 0.33 | 0.41 | 0.81 | 0.419 | -0.48, 1.15 |
| | Group | -0.60 | 0.40 | -1.49 | 0.137 | -1.39, 0.23 |
| | Group x time | 0.64 | 0.58 | 1.10 | 0.274 | -0.51, 1.79 |
| | Eczema | | | | | |
| | Time | -0.80 | 0.57 | -1.41 | 0.161 | -1.92, 0.32 |
| | Group | -0.19 | 0.56 | -0.33 | 0.742 | -1.29, 0.92 |
| | Group x time | 0.50 | 0.80 | 0.62 | 0.537 | -1.10, 2.09 |
| Migraine | | | | | | |
| | Migraine | | | | | |
| | Time | 0.23 | 0.40 | 0.58 | 0.562 | -0.57, 1.04 |
| | Group | -0.32 | 0.40 | -0.81 | 0.420 | -1.11, 0.41 |
| | Group x time | 0.35 | 0.57 | 0.61 | 0.543 | -0.78, 1.48 |
| GI disorders | | | | | | |
| | Bloating | | | | | |
| | Time | 0.34 | 0.23 | 1.46 | 0.148 | -0.12, 0.81 |
| | Group | -0.33 | 0.23 | -1.38 | 0.168 | -0.79, 0.14 |
| | Group x time | 0.32 | 0.33 | 0.97 | 0.332 | -0.33, 0.98 |
| | Abdominal pain | | | | | |
| | Time | 0.22 | 0.29 | 0.77 | 0.443 | -0.35, 0.80 |
| | Group | -0.22 | 0.28 | -0.78 | 0.434 | -0.79, 0.34 |

| | | | | | |
|---|---|---|---|---|---|
| Group x time | 0.17 | 0.41 | 0.41 | 0.684 | -0.65, 0.98 |
| Burning | | | | | |
| Time | 0.58 | 0.33 | 1.74 | 0.086 | -0.08, 1.24 |
| Group | 0.94 | 0.34 | 2.76 | 0.006 | 0.27, 1.62 |
| Group x time | -1.01 | 0.48 | -2.12 | 0.037 | -1.95, -0.06 |
| Flatulence | | | | | |
| Time | 0.24 | 0.27 | 0.91 | 0.366 | -0.29, 0.82 |
| Group | -0.33 | 0.28 | -1.20 | 0.231 | -0.87, 0.21 |
| Group x time | 0.36 | 0.38 | 0.93 | 0.356 | -0.41, 1.12 |
| **Bristol scale (1–7)** | | | | | |
| Time | -0.00 | 0.20 | -0.01 | 0.991 | -0.40, 0.40 |
| Group | 0.19 | 0.20 | 0.99 | 0.325 | -0.19, 0.58 |
| Group x time | -0.10 | 0.28 | -0.36 | 0.723 | -0.66, 0.46 |

Note: Group coded as 0 = Placebo, 1 = DAO.

**Table S3.** Fixed effects for the mixed linear model examining the clinical symptoms according to responders

| | | Estimate | SE | *t* | *p*-value | 95% CI |
|---|---|---|---|---|---|---|
| **Sleep** | | | | | | |
| | **Sleep quality** | | | | | |
| | Time | -0.42 | 0.64 | -0.67 | 0.507 | -1.71, 0.86 |
| | Group | -0.42 | 0.46 | -0.91 | 0.366 | -1.35, 0.50 |
| | Group x time | 0.37 | 0.69 | 0.54 | 0.594 | -1.03, 1.78 |
| **Atopic dermatitis** | | | | | | |
| | **Dry skin** | | | | | |
| | Time | 0.43 | 1.02 | 0.42 | 0.678 | -1.64, 2.49 |
| | Group | 0.62 | 0.69 | 0.90 | 0.370 | -0.76, 2.01 |
| | Group x time | -0.58 | 1.12 | -0.52 | 0.608 | -2.85, 1.69 |
| | **Hives** | | | | | |
| | Time | 0.42 | 1.06 | 0.40 | 0.691 | -1.71, 2.56 |
| | Group | -0.15 | 0.79 | -0.20 | 0.846 | -1.71, 1.41 |
| | Group x time | -0.04 | 1.16 | -0.04 | 0.970 | -2.38, 2.30 |
| | **Eczema** | | | | | |
| | Time | 0.77 | 1.79 | 0.43 | 0.669 | -2.83, 4.36 |
| | Group | 0.46 | 1.35 | 0.34 | 0.736 | -2.23, 3.14 |
| | Group x time | -1.68 | 1.93 | -0.87 | 0.388 | -5.58, 2.21 |
| **Migraine** | | | | | | |
| | **Migraine** | | | | | |
| | Time | 0.57 | 1.09 | 0.52 | 0.606 | -1.64, 2.77 |
| | Group | 0.28 | 0.80 | 0.36 | 0.723 | -1.30, 1.87 |
| | Group x time | -0.34 | 1.19 | -0.29 | 0.776 | -2.76, 2.07 |
| **GI disorders** | | | | | | |
| | **Bloating** | | | | | |
| | Time | 0.86 | 0.63 | 1.36 | 0.180 | -0.41, 2.13 |
| | Group | 0.74 | 0.47 | 1.55 | 0.124 | -0.21, 1.68 |
| | Group x time | -0.61 | 0.69 | -0.88 | 0.383 | -2.00, 0.78 |
| | **Abdominal pain** | | | | | |
| | Time | 0.29 | 0.77 | 0.37 | 0.714 | -1.28, 1.85 |
| | Group | 0.19 | 0.56 | 0.34 | 0.734 | -0.93, 1.31 |
| | Group x time | -0.03 | 0.85 | -0.08 | 0.940 | -1.77, 1.64 |
| | **Burning** | | | | | |
| | Time | 1.71 | 0.69 | 2.47 | **0.018** | 0.31, 3.12 |
| | Group | 1.26 | 0.52 | 2.43 | **0.017** | 0.23, 2.29 |
| | Group x time | -1.34 | 0.76 | -1.76 | 0.086 | -2.88, 0.20 |
| | **Flatulence** | | | | | |
| | Time | 1.00 | 0.56 | 1.77 | 0.084 | -0.14, 2.14 |

| | | | | | |
|---|---|---|---|---|---|
| Group | 1.09 | 0.42 | 2.62 | **0.011** | 0.26, 1.92 |
| Group x time | -0.89 | 0.62 | -1.44 | 0.157 | -2.14, 0.36 |
| **Bristol scale (1–7)** | | | | | |
| Time | 0.14 | 0.49 | 0.30 | 0.769 | -0.84, 1.15 |
| Group | 0.14 | 0.37 | 0.39 | 0.699 | -0.59, 0.87 |
| Group x time | -0.17 | 0.54 | -0.31 | 0.758 | -1.25, 0.91 |

Note: Group coded as 0 = Non responders, 1 = Responders.
